# Treatment of the Diseases of the Eye by Means of Prussic Acid Vapour, and Other Medicinal Agents

**Published:** 1843-04

**Authors:** 


### Art. XVI.

*Treatment of the Diseases of the Eye, by means of Prussic Acid Vapour, and other Medicinal Agents.* By A. Turnbull, m.d.—*London,* 1843. Small 8vo, pp. 89.

The author of the small work before us has, for several years past, been made known to the public and the profession, through the medium of his own writings and the writings of his friends, in pamphlets, journals, and newspapers, as one of the most distinguished members of that gifted and benevolent brotherhood whose delight and glory it is to redeem the lost dignity of physic, by curing the incurable. We take shame to ourselves that we have so long deferred to give, in our pages, a more permanent record of his merits,—a fault for which we shall now, in our humble way, make amends, by inscribing the name of Turnbull on our scroll of immortals, already resplendent with those of Curtis, Yearsley, Dickson, &c. As opportunity offers, we shall hope to heighten the glories of this galaxy of medical miracle-workers, by adding those stars of rival magnitude—the Cronins, Courtenays, Gosses, Ramadges, Van Butchels, &c. &c., which have been so long shedding benignantly their "bright effluence" on a suffering public and a benighted profession.

Like the great Curtis, Dr. Turnbull has built his fame on the double basis of the Eye and Ear; and like him also, and like another man of kindred genius, William Shakspeare, he has

> "Exhausted worlds, and then imagined new."

It is, doubtless, known to all our readers, that, two or three years since, Dr. Turnbull, having restored the hearing of the few deaf persons still left in England after the labours of Curtis and Yearsley, betook himself to Scotland, and, in a single tour in a single season, annihilated deafness as a disease, in that extensive and populous country. The history of this triumphant "Progress" of the great Ear-doctor, and the immediate results of his treatment, are recorded in Chambers's Edinburgh Journal; and the final results were made known at a subsequent period, by Mr.

Kinniburgh, the excellent and intelligent Head Master of the Institution for the Deaf and Dumb in Edinburgh.

From the work before us it would seem that Dr. Turnbull has now set about doing that for the Eye in England, which he did for the Ear in Scotland; and in the present notice we shall endeavour to do for him in the south, what Mr. Chambers did for him in the north,—viz., blazon his miracles in their very dawn; not doubting that some London Kinniburgh will hereafter do himself the honour to lay them before the public in their meridian glory—their complete development, and final effects.

Although the number of the miraculous cures recorded in this book is not great,—the author having only been able to "appropriate a few spare minutes to its preparation, amid the multiplicity of professional duties,"—we presume that Dr. Turnbull has nearly exhausted the stock of blindness in London, as we perceive from an advertisement appended to his work, that he is already turning his disinterested look "to fresh fields, and pastures new."

"*Preparing for publication,*—SUGGESTIONS FOR AN IMPROVED TREATMENT OF THE DISEASES OF THE HEART AND LUNGS."

*Improved Treatment! Diseases of the Heart and Lungs!* Good heavens! this must surely be a mistake. Where has he met with such diseases? What will Dr. Ramadge say to his illustrious brother, for even hinting to the world that there *can* be an "improved treatment," or even that any such "curable" diseases yet remain to be cured? For our own parts, we have not seen any case of the kind since the week after the last edition of Dr. Ramadge's book was published; and we are told that Mr. Farr has, in his recent Report, omitted the very title of such affections from his list of mortal diseases.

Dr. Turnbull being an illustrious member of that class of great men, of one of whom it was long ago said,

"None but himself can be his parallel;"

and as we sincerely believe that his deserts are of that exalted sort,

"That those who paint them truest praise them most;"

we shall, on the present occasion, do nothing more than we recently did in the analogous case of the great Dr. Dickson, viz., give a simple abstract of the singular doctrines, and still more singular facts, which are recorded in the volume on our table. We are sure that on perusing this abstract, our readers will be of opinion that all comment is unnecessary, as the said doctrines and facts must, in their minds, "plead, like angels, trumpet-tongued," in behalf of the instantaneous installation of Dr. Turnbull into the illustrious band above commemorated, who, with him, are patiently working for, and calmly awaiting, that Apotheosis which is now enjoyed by their great precursors—the CAGLIOSTROS and KATTERFELTOS of the last age, and the ST. JOHN LONGS and MORRISONS whose mundane glories we ourselves have had the privilege to witness.

We may, first of all, direct our readers' attention to some of Dr. Turnbull's theories, which, to say the least of them, are original. One of them meets us at the very threshold. "It is a well-known fact," says Dr. T., "that the eyes of those who have been destroyed by hydrocyanic acid show none of the usual symptoms of dimness for a length of time after death. On the contrary, the eye is clear, and the pupil much dilated." This we believe to be true, and generally known; but is it possible to conceive anything more original and ingenious than the conclusion Dr. T. draws from the glistening, staring expression of the eyes of one poisoned by prussic acid? "This satisfied me," he says, "that the acid exerted a specific action upon the eye, which might be made available as a medical agent for relieving many of the diseases to which that organ is so subject." (p. 4.) Now, as it happens that those who die from the influence of carbonic acid, and those who expire in a fit of epilepsy, present the same appearance of the eyes as that described by Dr. T., surely it ought not to expose one to be considered a follower of Thessalus to conclude, in the same analogical mode of reasoning, that a brazier of charcoal, not pushed too far, or a gentle epileptic fit, might cure a cataract or an amaurosis. Good observations are undoubtedly the basis of all our reasoning, but it is plain that even the most striking facts may remain unapplied, till they are seen by the eyes of genius. "Take, thou, my eyes, and see!" said Nicomachus; and so may exclaim Dr. Turnbull, on the present occasion.

The following is another specimen of Dr. Turnbull's original mode of theorizing. Speaking of bisulphuret of carbon, and chlorocyanic acid, he says, "the action of these medicines, which contain so large a share of carbon, arises from the carbon in the vapour permeating the cuticle, and coming in contact with the oxygen in the vessels." (p. 12.) The same theory is again referred to, when treating of the utility of oily embrocations, applied to the forehead. "The rationale, in my opinion," says Dr, T., "is, that the large quantity of carbon in some of the essential oils, and its solubility in alcohol, permits it, by the friction, to pass through the cuticle and unite with the oxygen." (p. 71.) At first glance we felt rather disposed to quarrel with Dr. Turnbull, for introducing so commonplace a topic as the cure of amaurosis by stimulating embrocations, but a moment's reflection on the lucid and most satisfactory theory of their *modus operandi*, as well as that of the bisulphuret of carbon and the chlorocyanic acid, made us change our views. The effect of stimulating liniments was formerly attributed to their action on the nerves of the skin, but how much more satisfactory is Dr. T.'s explanation! It is the carbon, permeating the cuticle, and uniting with the oxygen in the blood, which, by "forming carbonic acid, evolves heat in the ratio of the quantity consumed by the oxygen (p. 12), and cures the disease. We are only sorry that Dr. T. has nowhere given us the proofs that the bisulphuret of carbon, and the other substances he mentions, are decomposed by being brought into contact with the cuticle. This is, however, a trivial omission, and, no doubt, to be ascribed to the hurry into which the author has lately been thrown by his "multiplicity of professional duties." What would he say, to extending his theory of those carbon-containing substances a little farther? As the eyes of those who have been destroyed by carbonic acid show none of the usual symptoms of dimness for a length of time after death, may not substances which yield carbon to the oxygen in the vessels, when applied to the cuticle or to the conjunctiva, exert a specific action upon the eye? He has been so successful as to prussic acid, that we are tempted to submit this hint to his better judgment, as a further extension of the principle of which he is undoubtedly the original discoverer.

As another example of Dr. Turnbull's wonderful therapeutical science, we may quote what he says regarding the action of the vapour of concentrated prussic acid on the eye. He tells us, that he puts into an ounce phial a drachm of the acid, and holds it in close contact with the eye, the eyelid being open, for the space of about half a minute, or until the patient feels a little warmth, or the person holding the phial sees the pupil greatly dilated, and the vessels of the eye injected with blood. "The patient," says he, "is not sensible of pain from this peculiar state being induced, which appears to me to result from the powerfully sedative influence of the acid, thereby showing that two opposite powers—to wit, the stimulating and the sedative—are exerted at the same time; and thereby the uneasiness arising generally from a stimulant alone is prevented. Its greatest power in removing these diseases chiefly arises from the two powers being so blended, and thus enabling the eye to bear a sufficient stimulating action without injury." Who shall dare to deny, that the discovery of this double working of the vapour, stimulating and depressing, blowing hot and cold, not merely with the same breath, but at the same instant, is perfectly original and highly ingenious? To object to the author's statement, that it will be difficult for the bottle-holder to see the pupil dilating through the phial, or to hint that the equality of the two forces will be apt to render both of them null, would only show the objector's ignorance, and display the envy and malignity of spirit with which all new and wonderful discoveries are apt to be received.

We must now turn to Dr. Turnbull's statements regarding the practical results arising from the use of the medicinal agents he recommends. To begin with those which deviate least from ordinary practice: Dr. Turnbull cures amaurosis by stimulating embrocations applied to the forehead and temples. The stimulants he employs are chiefly the essential oils of cloves and pimento. But to show how impossible it is for a mind of genius to take up even the most ordinary matter without irradiating it with some new idea, Dr. Turnbull recommends those oily liniments to be sometimes, for a change, mixed with magnesia, made into pills, and administered internally. To balance accounts, castor oil, which is generally swallowed as a purgative, Dr. T. drops into the eye, with astonishing effect, in removing specks of the cornea, and otherwise purging the visual orb.

Dr. Turnbull's grand remedy, however, is the vapour of concentrated prussic acid. He favours us with fourteen cases, illustrating its effects in specks of the cornea, staphyloma, atrophy of the eye, inversion of the eyelashes, conical cornea, amaurosis, and cataract.

The *first case* is one of opaque corneæ, from variolous ophthalmia. The left eye was very much diminished in size, and the cornea studded with white spots. The right cornea was so opaque that neither iris nor pupil was to be seen. After four months' use of the vapour, the left eye had got tolerably clear, and the right was becoming transparent. The only remark a critic could make on this case respects the statement that the right eye will soon be the better eye of the two. The thoughtless reader may fancy that it was so from the commencement of the treatment, as the left eye was atrophic; but surely Dr. Turnbull and his patient know best.

The *second case* is one of disorganization of the eyes from inflammation, probably scrofulous. Dr. T. found all the humours of the left eye lost, a state of matters which, we believe, yields only to the prussic acid vapour. The right eye was much diminished in size, and the cornea very opaque. Under the use of the vapour, for a month, the chambers of the left eye became filled, to the extent of half the size of a healthy eye. The right cornea became almost transparent, and the sight almost perfect. Now we know perfectly the remark which the enemies of Dr. T. will make on this case. The age of the patient being thirteen, they will tell us that this is a period of life when the effects of scrofulous ophthalmia are apt suddenly to disappear. In reply, the doctor has only to ask them, when they saw any natural cure of this sort accomplished, where the humours were entirely lost, aqueous, crystalline, and vitreous, and the eyeball reduced to a mere stump, formed of its shrunk tunics? No! no! restoration, under such circumstances, can be effected only by the vapour of Russell Square.

The *third case* is one of staphyloma of the left eye, with such opacity of the right cornea that no appearance of iris or pupil could be discovered. Under the use of the vapour, the cornea of the right eye became transparent, and the opacity of the left was "so far removed as to give evidence that a continuance of the medicine will render it again perfect." Now, this statement some snarlers will perhaps be for rejecting as untrue. They will deny the possibility of an eye becoming perfect, in which the iris is united to an opaque and projecting cornea, or rather an opaque and projecting cicatrice occupying the place of the cornea, and that either by natural or by artificial means: they will tell us that the iris can never be separated, so as to allow the pupil again to appear; that the opaque cicatrice can never become transparent; and that consequently vision can never be recovered. Against all such assertions and aspersions Dr. T. will, no doubt, stand unmoved.

The subject of the *fourth case*, a girl of fifteen, was affected with opacity of the left cornea, and atrophy of the right eye. She was perfectly blind. Under the vapour, the left cornea became so clear that the patient walked alone.

The subject of the *fifth case*, a man, becomes blind, to use Dr. T.'s phrase, from ophthalmia; or, in other words, the corneæ become opaque, and the eyes are intolerant of light. Being blind does not mean, in Dr. T.'s language, that the retinæ are insensible, but only that the person cannot use his eyes to any purpose. All the applications used at the Eye Institutions where this patient attended, were of no use; such as black ointment, leeches, issues, steel internally, cupping, aperients, &c. Dr. T. applies merely two drops of warm almond oil to each eye. The man feels as if in another world, the intolerance is so much relieved. Castor oil is next used to the eye, to remove the opacities, and the cure is finished by the vapour.

The *sixth case* is one of choroid staphyloma of the left eye, and opacity of the right cornea, from ophthalmia neonatorum. Castor oil, and oil of almonds, diminish the size of the eyes; and the opacity of the right cornea is lessened by the same means. As the opacity clears, the pupil becomes visible, and an anterior capsular cataract is discernible. These effects of castor oil, and almond oil, are fully as wonderful as any of those of prussic acid and vapour, related by Dr. T. Prussic acid vapour, which, in case second, was used to make the eye grow, is now employed to make the eyes little, and the left eye diminishes to about half its former size. Light becomes visible to it, and the right cornea becomes transparent.

The *seventh case* is one of staphyloma of both eyes. The right had been operated on five times at an Ophthalmic Institution, but what kinds of operation had been performed, Dr. T does not say. Both eyes were perfectly opaque, studded with blue spots, having no appearance of iris or pupil, and stood prominently out beyond the lids. In the space of a fortnight, each eye became greatly diminished, and, as the opacity cleared off, a large triangular pupil made its appearance at the bottom of the right iris, enabling the patient to see. It is to be regretted that the treatment is not given, so that it is uncertain whether this artificial pupil was the effect of castor oil or the prussic acid vapour.

The *eighth case* is one of trichiasis and opaque corneæ. At one of the Eye Institutions at which the patient had attended, the upper eyelid had been cut off. After what we have learned of the effects of the vapour in atrophy of the eye and staphyloma, it is but a trifle for it to make the eyelashes resume their natural direction. Whether the amputated eyelid grew again under the application of the vapour, the doctor does not inform us. Case sixth shows the utility of castor oil, and almond oil, in reducing the size of too big an eye. Might we venture to inquire whether Dr. T. has tried *Macassar oil*, as a remedy likely to make little eyes to grow? or, at least to restore lost eyelids?

*Case ninth* is one of conical cornea, a disease which we have never known cured by any application, internal or external. It yields to Dr. T.'s application of castor oil to the eye, but still the sight is short, requiring the use of concave glasses. The vapour is employed, and the myopia is removed. As the number of short-sighted people in the world is pretty considerable, the vapour will no doubt soon have a fair trial. As it removes the myopia consequent to conical cornea, it will readily cure it where no such prominent change in the form of the eye has existed. Alas, for the spectacle-makers!

*Case tenth* is one of traumatic cataract of the right eye, with sympathetic inflammation of the left. Under the vapour, the cataract is half absorbed, and the amaurotic state of each retina yields, so as to allow the patient to read with the left eye, and to begin to see with the right. Dr. T. thinks, that unless the capsule of the lens had been lacerated in the accident which befel the right eye, the vapour could not have dissolved the cataract. But we here venture, with all deference, to differ from him. After the cures already related, there is nothing which may not be accomplished by the vapour. Extirpate the eyeball, apply the vapour, and to a certainty a new eye will begin to form, with which the patient will soon be able to read the smallest type. Should it threaten to grow too big, touch it with castor oil from time to time.

The *eleventh case* is another of traumatic cataract. The vapour is applied, and in three or four parts the cataract appears as if operated on by a couching needle. Here, again, the doctor's modesty overcomes him. "I am certain," says he, "it [the vapour] will not be a general remedy for lenticular cataract (unless employed in its incipient state), without operation, unless aided by rupturing the capsule of the lens by the needle first;" which is, in English, that the vapour will not cure cataract without operation, unless with operation, a proposition which an ignorant, or ill-natured critic might pronounce to be nonsense. But nobody knows better than Dr. T. what it is to cure a cataract without operation, with operation; unless, perhaps, some of his patients.

*Case twelfth* is one of amaurosis of the right eye, with fixed pupil. The vapour makes the pupil act, but whether it makes it expand, as formerly stated at p 5, or contract, is not said. Vision returns.

*Case thirteenth* is one of incomplete amaurosis of both eyes. The right eye recovers, so that the patient can thread her needle. Upon the pupil of the left eye the vapour had not the least effect, which leads Dr. T. to observe that "when prussic acid does not dilate the eye, in amaurosis, much good is not to be expected from its use. In such cases," he adds, "I am inclined to believe that the disease is caused by pressure upon the optic nerve;" thus overthrowing the dogma, that such pressure is the cause of dilatation of the pupil.

The *fourteenth case* is one of total blindness from birth. The eyes were sunk in the orbits, and the pupils immoveable. The doctor begins by making the eyes grow by castor oil. A monthly increase takes place, and the pupils manifest a disposition to act. The vapour is then applied. The eyes assume a healthy appearance, and the patient begins to distinguish objects.

Such is an abstract of the *facts*, related in this work. They have excited our wonder to the utmost. It would be base to descend from the contemplation of the grand discoveries of Dr. T. to a criticism of his style, which is sometimes a little confused, a thing not to be thought surprising, considering the sort of hydrocyanic atmosphere in which his work must have been composed.

To the use of the vapour of concentrated prussic acid timid patients, it would appear from the doctor's own testimony, have objected. How unlucky then is it, that substances of a less dangerous character answer nearly the same purpose! We are afraid that almond oil, and castor oil, to say nothing of Macassar, will be fearfully abused in the cure of sore eyes, now that the doctor has unfolded their extraordinary virtues.

---

